# Aspartame—True or False? Narrative Review of Safety Analysis of General Use in Products

**DOI:** 10.3390/nu13061957

**Published:** 2021-06-07

**Authors:** Kamila Czarnecka, Aleksandra Pilarz, Aleksandra Rogut, Patryk Maj, Joanna Szymańska, Łukasz Olejnik, Paweł Szymański

**Affiliations:** 1 Department of Pharmaceutical Chemistry, Drug Analyses and Radiopharmacy, Faculty of Pharmacy, Medical University of Lodz, Muszyńskiego 1, 90-151 Lodz, Poland; lopi12@gmail.com (A.P.); aleksandra.rogut@stud.umed.lodz.pl (A.R.); patryk.maj@stud.umed.lodz.pl (P.M.); aptekabranice@gmail.com (J.S.); l.olejnik07@gmail.com (Ł.O.); 2 Department of Radiobiology and Radiation Protection, Military Institute of Hygiene and Epidemiology, 4 Kozielska St., 01-163 Warsaw, Poland

**Keywords:** aspartame, artificial sweeteners, cancer, metabolism, neurodegeneration

## Abstract

Aspartame is a sweetener introduced to replace the commonly used sucrose. It was discovered by James M. Schlatter in 1965. Being 180–200 times sweeter than sucrose, its intake was expected to reduce obesity rates in developing countries and help those struggling with diabetes. It is mainly used as a sweetener for soft drinks, confectionery, and medicines. Despite its widespread use, its safety remains controversial. This narrative review investigates the existing literature on the use of aspartame and its possible effects on the human body to refine current knowledge. Taking to account that aspartame is a widely used artificial sweetener, it seems appropriate to continue research on safety. Studies mentioned in this article have produced very interesting results overall, the current review highlights the social problem of providing visible and detailed information about the presence of aspartame in products. The studies involving the impact of aspartame on obesity, diabetes mellitus, children and fetus, autism, neurodegeneration, phenylketonuria, allergies and skin problems, its cancer properties and its genotoxicity were analyzed. Further research should be conducted to ensure clear information about the impact of aspartame on health.

## 1. Introduction

Artificial sweeteners (ASW), also known as non-nutrient sweeteners (NNS), became popular during the world wars in response to lowered sugar production due to the agricultural crisis. At that time, saccharin was highly accepted as an alternative to sugar. The sweetening properties of saccharin were accidentally discovered in 1879 by Remsen and Fahlberg, when Fahlberg found his dinner bread became very sweet after forgetting to wash his hands after a whole day in the lab [1]. At the beginning of the 1950s, it was believed that replacing sugar with ASW was desirable to reduce the calorific value of food products: the rapidly developing confectionery and fast-food industry was accompanied by increasing obesity. Although the demand for dietary products continued to increase, saccharin gradually lost favor because of its bitter aftertaste. It was necessary to find an ASW with a new improved taste.

An important development was the appearance of cyclamate, which did not give a bitter aftertaste and could be used to sweeten nonalcoholic beverages. Unfortunately, in 1970, the Food and Drug Administration (FDA) banned its use in the USA due to suspicions of causing cancer. Interestingly, it has not been banned in other countries [2]. Following this, the invention of aspartame proved to be a breakthrough. Aspartame entered the market in 1981, when it was marketed as NutraSweet, and was free of any suspicions regarding carcinogenicity [3,4]. For the first time, dairy products could be described as dietary. Aspartame remains a key sweetener in North America, Europe and Asia to this day [5].

Aspartame was invented in 1965 by James M. Schlatter. Schlatter obtained this compound as part of research into antiulcer drugs. He discovered the sweetness completely by accident, after licking it off his finger, against work safety regulations. After initial obstacles to the legalization of this compound as a food additive, its large-scale production began in 1981. The attractiveness of aspartame as a sweetener is since it is about 200 times sweeter than sugar, while its calorific value, at the concentrations giving the impression of sweetness, is almost zero. However, the taste of aspartame is not identical to that of regular sugar: the flavor takes longer to appear, and typically has an aftertaste. According to the FDA, the acceptable daily intake of aspartame for humans is 40 mg/kg bodyweight in Europe and 50 mg/kg bodyweight in the United States for both adults and children [6].

As many products contain aspartame, both children and adults can unintentionally consume larger amounts than those recommended by the FDA, which may lead to serious health complications [7]. Food with aspartame must be labeled with the information: “contains phenylalanine”. In addition, the labeling of foods containing aspartame must indicate that they are not recommended for cooking and baking [8]. Although many studies have been performed to determine the health effects of aspartame, the results of its long-term use remain difficult to predict and its use in pharmaceutical and food products remains controversial [9].

## 2. Aspartame Metabolism

Aspartame consists of two amino acids (L-phenylalanine and L-aspartic acid). It is hydrolyzed and absorbed in the gastrointestinal tract (GI) through the action of esterase and peptidases. Digestion releases methanol (10%), aspartic acid (40%) and phenylalanine (50%) (Table 1), which are absorbable in the intestinal mucosa [10]. These metabolites can be harmful at high doses and hence prolonged aspartame consumption may be a risk factor [11,12]. Indeed, the metabolism products of aspartame are believed to be more toxic than the original substance itself [13,14]. Methanol is firstly oxidized in the liver to formaldehyde and again to formic acid; however, while methanol is known to damage the liver, formaldehyde and formate are also responsible for the destruction of liver cells. In addition, during the process the formation of superoxide anions and hydrogen peroxide occur, which lead to protein denaturation and subsequent enzymatic changes [15,16,17]. According to the study on the impact of aspartame administration on trans-sulfuration pathway, decrease of most metabolites of the trans-sulphuration pathway in the liver was observed during experiment. Levels of cysteine, homocysteine, S-adenosyl-homocysteine, and S-adenosyl-methionine were increased. There was no significant change in methionine and cystathionine level [18]. All mentioned aspartame metabolites are toxic to the brain. Furthermore, rhenylalanine is mainly metabolized to tyrosine and smaller amounts of phenylethylamine and phenylpyruvate, while aspartic acid is metabolized into alanine and oxaloacetate [10]. It has been suggested that in human beings consuming large amounts, aspartame may be a significant source of formate, which can contribute to serious physiological changes. The role of aspartame in several disorders affecting human body remains to be investigated. Most importantly, people with phenylketonuria, a genetic disorder in which patients cannot convert phenylalanine to tyrosine, must avoid aspartame. Due to the harmful effects of aspartame on phenylketonuria patients, according to the FDA requirements all products containing aspartame must have a label informing about the presence of phenylalanine [19].

## 3. Properties and Application in Food and Pharmaceuticals

Aspartame has also been used in the encapsulation process to improve the properties of the payload, imparting greater stability, and improving taste, color and texture and extending storage times [20]. This technique can be used not only in food industry, but also in cosmetics, agrochemical industry and pharmaceutical industry to control the release of the drug [21]. An encapsulated substance is enclosed within a carrier material, which forms a protective shell. In the case of the food industry, this protective shell must be an inert substance must be food-grade, biodegradable and able to form a barrier that separates the internal phase from the surroundings. Encapsulates can be prepared by spray-drying, spray-chilling, freeze-drying, melt extrusion and melt injection [22]. In addition, encapsulation can take place by nanoencapsulation, in which nanoscale particles (1- to 1000 nm) are produced, or microencapsulation, with larger particles (1 to 1000 um). This difference in size is significant: nanoencapsulated bioactive compounds have higher bioavailability, and their release can be more precisely controlled. Microencapsulation is usually used to protect substance from the influence of the surroundings, and to produce sustained- or prolonged-release drugs [21].

The use of aspartame in food producing is limited due to its instability: although it is stable in solid form, it is degraded in solution, especially in high temperatures or pH values above 6. Aspartame also loses its sweetness after reacting with several flavorings, aldehydes or ketones. Hence, there is growing interest in more encapsulated compositions of aspartame [23]. The structure of aspartame (carboxylate and amino groups) allows it to bind metals, and can chelate heavy metals from food, drinks and the packaging in which these products are served and stored [20].

Aspartame is also decomposed by baking. However, it is possible to protect aspartame salts ((APM.H)_2_SO4, (APM.H-)SO_3_CH_3_) and its metal complexes (Mg^2+^APM-2Cl, Fe^3+^APM-3Cl, Al^3+^APM-3Cl, Ca^2+^APM-2Cl, Zn^2+^APM- 2Cl) by fats, thus allowing its use in pastries [23]. Due to its high sweetness, aspartame is commonly used in comestible products such as chewing gums. As its sweetness is easily degraded in the presence of moisture, or during storage, various substances are used to encapsulate it, for example, shellac, Zeuin, agar, alginates, ethyl cellulose, hydroxpropylmethyl cellulose, dextrin, gelatine and modified starch. Gums treated with encapsulated aspartame remain sweet for longer and the flavor is released during chewing [24].

## 4. Obesity

The world is experiencing an obesity pandemic. According to the World Health Organization (WHO), over one billion adults throughout the world are overweight, i.e., with a body mass index (BMI) ≥ 25 kg/m^2^. Of these, at least 300 million are considered obese (BMI ≥ 30) [25]. Overweight and obesity are associated with numerous comorbidities of great public health concern, including hypertension, cardiovascular disease, diabetes and depression, as well as breast, endometrial, colon, and prostate cancers [26,27]. As a result of the aggressive marketing of food industry companies, the role of artificial sweeteners has changed from sugar substitutes to health substitutes. Foods treated with ASWs are sold as healthy alternatives to sugar, in particular for the diabetic population and people trying to keep fit. Although these claims seem promising, there has never been a sufficiently large response to this topic. Another serious problem with artificial sweeteners is their unregulated use. There is some research that the intake of artificial sweeteners by children aged 6–10 years exceeded recommended daily intake (ADI) by 54% [28].

Other studies suggest that replacing sugar-sweetened beverages with artificial sweeteners has no influence on total energy intake and can increase future consumption [29,30]. These studies also showed that the type of sweetener has no influence on appetite and hunger ratings. Participants compensated for the energy deficit resulting from the replacement of caloric sweeteners with artificial sweeteners. What is more, there is some evidence that the flavor of both sugar-sweetened and artificially sweetened drinks increases subjective hunger and therefore energy intake and weight gain too. These issues need further investigation [31,32].

Unfortunately, assessing the influence of artificial sweeteners on the growth in the number of obese people is very difficult. For some people, eating foods that are dietary justifies the consumption of excess calories from other food sources. Therefore, it is difficult to clearly determine whether the occurrence of obesity is specifically associated with the consumption of products containing artificial sweeteners (including aspartame), or simply with too many calories [33,34].

## 5. Diabetes Mellitus

Aspartame has also been blamed for causing type 2 diabetes mellitus (T2DM) and may not meet the expectation of being healthy alternative to sugar in sweetened beverages [35]. A recent study investigating the effect of sucrose-sweetened and artificially (aspartame) sweetened food and drinks on inflammatory markers in overweight subjects found that consumption of sweetened items significantly increased plasma haptoglobin and transferrin but did not significantly increase the C-reactive protein level. This study corroborates findings from others that suggest that the pro-inflammatory process underlying the greater risk of diabetes may be exacerbated by a high intake of rapidly digested and absorbed carbohydrates and artificial sweetener [36].

Unfortunately, the link between non-nutritive sweeteners (NNS) and T2D risk remains unclear, and the relationship is complicated by factors such as obesity or intestinal microflora. The true relationship between NNS and metabolic disease is unclear [37]. Many of the available studies could not be considered reliable, as the criteria for inclusion of patients were not clear and risk factors for diabetes were not included [38,39]. It has not been confirmed that NNS intake is directly associated with T2D, or whether it has favorable or harmful effects on the disease; no solid conclusion has been drawn in systematic reviews and meta-analyses due to the wide range of study types and designs. Well-designed studies are urgently needed for covering all types of potentially interfering factors and those which play a role in T2D development [40].

## 6. Impact of Aspartame on Children and Fetuses

A healthy diet is particularly important during pregnancy to allow the proper development of both mother and fetus [41,42]. Many prospective cohort studies note a relationship between artificial sweeteners (ASWs) consumption and putting on weight in children [43,44,45]. Low-calorie sweeteners (LCS) are regarded as safe to consume during pregnancy and lactation if recommended levels are not exceeded [9,46,47,48,49]. However, some reports have associated preterm delivery and allergic diseases in offspring with consumption of ASWs in beverages [50,51,52]. Despite this, a review of papers found no such association between ASW and preterm delivery [53]. While a recent study found aspartame metabolism products to cross into the placenta [46], a dose of 200 mg/kg of bodyweight of aspartame, i.e., four to five times higher than recommended daily intake, was not found to result in methanol poisoning or mental retardation caused by increased Phe level in the blood of offspring [54], suggesting that consumption within recommended levels should not cause adverse effects to the fetus [46,49]. Another study on fish embryos from medaka (Oryzias latipes) found that aspartame treatment increased embryo heart rate, gently decreased head growth, and caused anxiety-like behavior; interestingly, aspartame in combination with caffeine demonstrated a lower increase than caffeine alone and caused advanced eye development and decreased hatchling body length. The authors ranked aspartame in second place for developmental toxicity [55].

A study on the impact of consumption of low doses of aspartame and stevia with obesogenic diet on rat dams and their offspring found that consumption of aspartame during lactation and gestation by rat dams increased obesity in offspring at weaning and had a detrimental effect on glucose and insulin tolerance, which only occurred in male offspring. In addition, the intestinal microbiota of the offspring was altered, and aberrations were observed regarding gene expression in the mesolimbic reward system. This research concluded that aspartame consumption had a greater impact on male offspring, associated with higher susceptibility of male offspring to maternal stress and diet during pregnancy [45,56]. Similarly, adult rats whose mothers consumed aspartame during pregnancy demonstrated greater weight gain and higher plasma glucose, LDL and total cholesterol than controls, the results imply that prenatal exposure to aspartame can impair learning [44]. Such mice may also have an impaired system of satiety [12] and an unbalanced metabolism due to the increased Phe levels in the blood, which is transformed by other metabolic pathways [44,57,58]. Aspartame use has also been associated with increased risk of type 2 diabetes, cardiovascular diseases, nonalcoholic fatty liver disease and hormone-related cancers [59,60,61,62,63,64,65,66]. Studies also indicated elevated risk of early menarche among girls aged 9–10 years. (<11 years old) [64].

## 7. Genotoxicity

Although the safety of use of aspartame has been assessed by the Joint FAO/WHO Expert Committee on Food Additives (JECFA), the US Food and Drug Administration (FDA) and the European Food Safety Authority (EFSA), among others, few studies have tested its genotoxic properties in vivo and in vitro [67,68,69,70,71,72,73,74]. Unfortunately, none of these studies were conducted according to GLP [74,75]. Despite the lack of pharmaceutical studies in the GMP system, publications state that aspartame is not genotoxic [68,71,72,75,76]. In addition, research techniques have developed over the years and current methods are more accurate and reliable [75].

The breakdown of aspartame results in formaldehyde production through the conversion of methanol by ADH [77,78]. Formaldehyde may damage DNA by its ability to crosslink proteins with it, and as aspartame is an exogenous sources of formaldehyde, its consumption may hence induce genotoxicity [79,80].

However, GLP-based studies on the genotoxicity and mutagenicity of aspartame based on bacterial reverse mutation and in vivo murine micronucleus testing found aspartame to have neither genotoxic nor mutagenic effects [75]. Other genotoxicity and mutagenicity studies were performed by Najam et al. with aspartamine alone and in combination with sitagliptin, which is an oral antidiabetic drug [81,82]. The results of the performed Comet assay were similar to the positive dose-dependent chromosome aberration test [67,82], while aspartame demonstrated significant mutagenic effects in Ames assay for TA100. In addition, aspartame with sitagliptin did not demonstrate greater effects than sitagliptin alone [82]. Unfortunately, opposite results have been obtained in more recent studies [75,82].

For industrial purposes, aspartame is often combined with other substances to obtain a desired taste. Genotoxicity studies found the combination of aspartame with another frequently used sweetener, acesulfame-K, to result in an increase in genotoxic activity conditioned by a dose-response relationship. Since the same studies also showed no genotoxic effects in acesulfame-K, it can be expected that it is aspartame that increases genotoxicity [68].

## 8. Behavioral Disorders

Aspartame is suspected of causing neurological and behavioral disorders in humans. It causes neuropsychiatric reactions such as headache, convulsions and depression [83]. In the body, aspartame is transformed into phenylalanine (Phy), aspartic acid and methanol. These metabolites can affect the neurochemical state of the brain and influence the level of neurotransmitters [12]. Phenylalanine is metabolized to tyrosine and to a lesser degree, to phenylethylamine and phenylpyruvate; aspartic acid is transformed into alanine and methanol, which is then transformed into formic acid via formaldehyde [84]. Neurotransmitters such as serotonin (5-hydroxytryptamine, 5-HT), norepinephrine (NE), and dopamine (DA) play a significant role in the regulation of mood, cognition, learning, motor activity, vigilance, reward, sleep, appetite and cardiovascular function [85]. The amino acids phenylalanine (Phy), tyrosine (Try) and tryptophan (Trp) determine the synthesis of NE, DA, and 5-HT [86]. Consumption of aspartame significantly increases plasma Phy, which competitively inhibits Tyr hydroxylase and Trp hydroxylase, the rate-limiting enzymes for dopamine and serotonin synthesis. The resulting fall in dopamine and serotonin levels in the brain has serious consequences, such as depression [87,88].

One component of aspartame, aspartate, is a biogenic amino acid that also acts as a neurotransmitter. It is classified as an excitatory amino acid; these which are needed to maintain normal brain function when balanced with inhibitory amino acids [89]. Disturbing this balance results in the development of mood disorders [90]. Aspartate and glutamate competes with each other to bind with N-methyl-D-aspartate (NMDA) receptors, it could increase calcium (Ca^2+^) influx and disturbed intracellular Ca^2+^ homeostasis, and, as a result neuronal cell functions were altered [91,92].

Aspartame also stimulates the sympathetic nervous system by causing an increase in cortisol steroid levels in the adrenal glands via the hypothalamic-pituitary-adrenal (HPA) axis (Table 2) [93,94]. It also changes the composition of the gut microbiota [95,96]. Most often, this results in long-term changes in behavior, as well as increased corticosterone release and adrenocorticotropic hormone (ACTH) level [76,97]. Cortisol activates various areas of the brain by suppression of hippocampal activation, enhancement of amygdala activity, and inside the prefrontal cortex, which affects psychological states that inform the people for preserving physiological homeostasis [98]. Aspartame is also responsible for causing mental stress [17,99].

The gut microbiota can produce various important neurotransmitters. For example, Escherichia, Bacillus and Saccharoyces spp produce NE; Candida, Streptococcus, Escherichia, and Enterococcus spp produce 5HT; Bacillus produces DA [100,101,102]. It is likely that these neurotransmitters induce molecules release from epithelial cells that in turn modulate neuronal signaling within the enteric nervous system (ENS), or act on primary afferent axons [103]. It is possible that aspartame can dysregulate the intestinal microbiome and disturb the production of neurotransmitters, resulting in neurobiological impairments.

## 9. Autism Spectrum Disorder (ASD)

Autism is a disease manifested by problems with communication and social interaction, some problems with movement, sensory disorders and repetitive behaviors can occur [104]. The rise in autism increased significantly in 1983 with the rise in consumption of aspartame in carbonated beverages, also known as diet soda [105], and has continued to do so as aspartame consumption has grown [106]. Autism has been observed more often among children whose mother was obese or suffered from diabetes, both of whom may demonstrate increased aspartame consumption [107,108]. In the process of digestion, 11% of the aspartame is transformed into pure methanol [109], which is known to be neurologic teratogen and poison for humans [77,110,111]. Methanol is metabolized in our body to formaldehyde by alcohol dehydrogenase class 1 (ADH) [72], and then oxidized to formic acid in the cytosol. When ADH is not available, formaldehyde can react with DNA and RNA; the products can cross link with basic proteins in the cytosol and inactivate them. Such changes have been found in the brains of people suffering from autism [106].

A study of the association between autism and dietary methanol, whose main source in the United States is aspartame [105,109], among mothers whose children were born after 1984 found that women who gave birth to children with autism consumed on average over twice the amount of dietary methanol each week than those who did not [109]. However, Parker et al. argue that although methanol liberated from aspartame is a toxin, causes oxidative stress, which is also associated with autism, and that its use is connected with a rise in ASD, aspartame itself does not trigger autism [109,112,113]. They note that previous uses of methanol, in canned vegetables for example, did not coincide with an incidence of autism, and that the removal of aspartame from Pepsi cola did not result in a decrease in the United States [113]. Although the breakdown product of aspartame is a potential teratogen and toxin [72,77,110], the studies conducted so far, do not provide conclusive results confirming that aspartame also exhibits the same properties [109,113].

## 10. Neurodegeneration Due to Long Term Use of Aspartame

Studies suggest that aspartame and its metabolites increase the risk of neurodegenerative diseases such as Alzheimer’s disease, Parkinsonism, multiple sclerosis and brain tumors [10,114]. Methanol, aspartame metabolite, causes increased levels of free radicals resulting in damage to the cell membrane, caused by peroxidation of fatty acid in the phospholipids, damage to cellular components such as nucleic acid lesions, as well as gene damage and repair resulting in apoptosis or necrosis. Moreover, aspartame activates various calcium channels in neurons resulting in cell death. In addition, elevated free radical levels decrease enzyme activity in the liver [115]. Aspartame intake also results in elevated H_2_O_2_ levels, placing added oxidative stress on cells [17]; this has been confirmed in studies recording elevated nitric oxide and lipid peroxidation levels after a 90-day diet with aspartame [116]. Mitochondrial oxidative stress leads to apoptosis of adrenal and brain cells. Long-term administration of aspartame has been found to result in degenerative changes in the sciatic nerves, including demyelination, disruption and splitting of myelin lamellae, lamellar structure deformation and myelin loop formation, as well as irregular thickening of myelin sheaths. In addition, other, less frequent axonal changes can be observed: axons can be shrunk, compressed and distorted, their mitochondria can be swollen, as well as RER dilatation and vacuolation of Schwann cell cytoplasm. Aspartame also appears to have a negative influence on the cerebral and cerebellar cortex (Table 3) [73].

Long-term use results in altered glutathione peroxidase and glutathione reductase activities [18] and has been found to affect the hippocampus. Its metabolites, aspartate, and methanol, also influence the signaling pathway associated with memory, resulting in neurodegeneration and memory loss. Aspartate acts as a glutamate agonist at the NMDA receptor. Calcium influx activates calcium-calmodulin-dependent protein kinase II (CaMKII). CaMKII induces cAMP response element-binding protein (CREB-P) pathway. Methanol inhibits phosphorylation. [117], while aspartate is an excitotoxic substance [78] which interrupts homeostasis and modifies neuronal cell function [10]. Persistent aspartame intake also results in a decrease in hippocampal acetylcholinesterase activity. All these mentioned factors affect learning skills and memory [115].

Aspartame is also amyloidogenic, being able to spontaneously assemble into an amyloid-like nanostructure, with more intensive aggregation observed at higher aspartame concentrations. The aggregation is characterized by the formation β-like structures. Moreover, existing aspartame fibrils can induce formation of amyloid-like structures from other molecules such as proteins and peptides. The structure formed by aspartame can initiate β-sheet aggregation in the Aβ1-40 peptide. This process leads to the formation of Aβ-amyloid fibrils, which are associated with Alzheimer’s disease [118].

Phenylalanine (Phy) is needed to synthesize tyrosine, dopamine, noradrenaline, and adrenaline. However, Phy demonstrates strong affinity to large neutral amino acid (LNAA) carrier systems, thus competitively inhibiting other amino acids which rely on this carrier system (tyrosine, tryptophan) and preventing them from crossing the BBB. After aspartame intake, plasma phenylalanine level is increased, thus influencing neurotransmitter concentration in the brain [10]. High levels of plasma Phy inhibit tyrosine and tryptophan hydroxylase, the latter of which is necessary for catecholamine synthesis. A decreased catecholamine level can lead to many disorders [114].

Aspartame can be identified as chemical stressor since after aspartame intake corticosterone and adrenocorticotropic hormone levels in plasma are elevated. Elevated adrenal cortical steroid level leads to decreased 5-hydroxytryptamine function and could contribute to a depressive state [17]. Moreover, elevation of corticosterone level significantly reduces the weight and size of spleen and thymus [116].

## 11. Allergies and Skin Problems

A four-year placebo-controlled clinical study published in 1993 found aspartame and its metabolites induce hypersensitivity reactions such as urticaria or angioedema on the same level as placebo. The adverse reactions after aspartame or placebo appeared with the same frequency at all doses [119]. A double-blind randomized crossover study in the United Kingdom compared a group of sensitive individuals with self-reported hypersensitivity and a group of nonsensitive individuals. The individuals were administered snack bars, either with or without 100mg of aspartame, with at least a seven-day break between administrations. No differences in the responses to either bar were found between sensitive and nonsensitive groups, indicating no adverse effects associated with acute ingestion of aspartame [120].

Some studies suggest that consuming aspartame could result in the development of skin problems. Systemic contact dermatitis (SCD) appears when a sensitive individual has contact with an allergen via cutaneous or systemic route. Aspartame can induce contact dermatitis, manifested by skin inflammation, and this has been attributed to an accumulation of formaldehyde, i.e., a metabolite of aspartame. However, daily aspartame intake must be huge to induce formaldehyde accumulation. Such effects have been observed in both adults and children. For example, in an 11-year-old patient who suffered from generalized erythema and eyelid dermatitis, discontinuation of an aspartame-containing drug mitigated both symptoms [121]. Similarly, in the case of 60-year-old patient with a six-month history of eyelid dermatitis, the symptoms completely resolved within one week of discontinuing an aspartame-based sweetener [122].

A formaldehyde-releaser is a substance that releases formaldehyde during its metabolism or under different condition. Well known formaldehyde releasers are quaternium-15, imidazolidine urea, diazolidine urea, DMDM hydantoin and 2-bromo-2-nitropropane-1,3-diol,tris(N-hydroxyethyl)hexahydrotriazine, formaldehyde resins [123]. It is possible to meet these substances in clothes, adhesives, paints, lacquers cosmetics, toiletries, and household products. After exposure, formaldehyde-sensitive individuals typically suffer from lesions of the mouth, eyelids and skin, as well as gastrointestinal disorders [124].

At concentrations of 25, 50 and 100 mg/kg aspartame has similar analgesic and anti-inflammatory properties to nonsteroidal anti-inflammatory drugs (NSAIDs), such as aspirin. At a concentration of 10 umkg-1, aspartame increases IL-10 production and decreases IL-6 production. Even though there is no proof that aspartame has anti-inflammatory effects, it may inhibit prostaglandin H2 synthesis and inhibit cyclooxygenase. Atopic dermatitis (AD) is a kind of inflammatory skin disease which is chronic and relapsing. AD is characterized by pruritus, dryness oedema, lichenification in skin lesions and erythematous. AD has a difficult pathogenesis comprising genetic, immunological, and environmental factors. It has been found that 0.5 um kg^−1^ and 0.5 mg kg^−1^ doses of aspartame inhibited ear swelling, one of the symptoms of AD and suppressed infiltration of eosinophils to skin lesions, degranulation, and epidermal thickening. T-lymphocyte level also has a considerable influence on the development of AD symptoms. Aspartame suppresses infiltration of CD4+T lymphocytes in skin lesions, and inhibits the formation of skin lesions by inhibiting synthesis of inflammatory cytokines caused by activated CD4+T. There are two phases during AD. In the first one, T helper type 1 dominates, and symptoms occur due to the production of IL-2 and IFN-gamma by Th1 cells, resulting in a chronic phase, and the development of hyperkeratosis and dermal thickening. In the second phase, T helper 2 (Th2) is dominant and produces IL-4, IL-5 and IL-13. This kind of AD manifests as an increased level of IgE and an accumulation of inflammatory cells in the skin lesions. Eventually, symptoms of chronic AD occur. Aspartame in concentrations of 0.5 μg kg^−1^ and 0.5 mg kg^−1^ alter serum IgE level [125].

## 12. Phenyloketonuria

Phenylketonuria (PKU) is a congenital recessive disease caused by changes to the gene encoding PAH (phenylalanine hydroxylase. When functioning correctly, PAH is in charge of the transformation of Phe into Tyr (tyrosine) [126]. However, in cases where PAH is deficient, phenylalanine accumulates in the blood and brain, leading to eczematous rash, motor deficits, irreversible intellectual disability, seizures, developmental problems, autism, microcephaly, aberrant behavior, and psychiatric symptoms. Sadly, the exact pathogenesis of brain dysfunction is not well known [127]. The symptoms can be avoided by implementing a low-Phe diet from birth; a simple test developed by Guthrie in 1960 allows the detection of phenylketonuria in newborns [128,129].

Following digestion, aspartame releases around 50% of its mass as pure phenylalanine [12,129,130]; following the consumption of 34mg/kg of bodyweight of aspartame by patients with moderate phenylketonuria, the level of plasma Phe rises to 16mmol/dL, compared to 11mmol/dL in healthy adults [131,132]. In addition, in healthy humans, the consumption of lower doses of aspartame (for example 10 mg/kg of bodyweight) results in an elevation of phenylalanine plasma levels from 4.5 mmol/dL to 6 mmol/dL, while in heterozygous phenylketonuria patients, this elevation is from 6.9 mmol/dL to 8 mmol/dL [133].

A UK study found many people suffering from phenylketonuria choose products without being aware of the amount of aspartame they contain; it is believed that the sugar tax in the country increases the use of sugar substitutes, including aspartame. In addition, approximately 25% of patients reported consuming aspartame with medications [134]. Studies have found aspartame and phenylalanine levels to vary between soft drinks; however, some consist of small amounts of APM and Phe, and could be safely consumed by patients with hyperphenylalaninemia and those treated with 2-(2-nitro-4-trifluoromethylbenzoyl)-1,3-cyclohexanedione (NTBC) (van Vliet et al., 2020).

## 13. Cancer Properties

One of the first and most serious allegations against aspartame consumption was its carcinogenic effect on the body; which has been attributed to its breakdown to harmful formaldehyde, with carcinogenic potential [135]. Consumption of 1 L of diet soda results in the transformation of approximately 600 mg of aspartame into 60 mg of formaldehyde [136,137,138], which greatly exceeds the ADI (0.15 mg of formaldehyde for every kg of body weight). There is clearly a great need for further studies to determine the impact of such aspartame metabolization.

Although aspartame was not found to result in the death of HeLa cells at any tested concentration, its presence contributed to an increase in the mRNA expression of the bcl-2 gene (anti-apoptotic gene). It also reduced the mRNA expression of the p53 tumor suppressor gene and the bax gene (apoptotic gene) in cancer cells. Expression of Ki 67 and PCNA (markers of proliferation) was significantly upregulated at both the mRNA and protein levels. Pandurangan et al. indicate that the influence of aspartame slows down the apoptosis process in cancer cells and increases their proliferation, which is a characteristic feature of cancer cells [139].

Other studies have found that aspartame may have a biological effect. A study of five-week-old, inbred CBA/CA (genotype of mice used in the study) female mice did not show any clear a dose-dependent pattern correlation, a relationship was observed only between the test and control groups. The test group demonstrated an increase in the expression of two oncogenes (c-myc, Ha-ras) and the p53 tumor suppressor gene in kidney, bone marrow and lymphoid tissues. However, a considerable elevation in the expression of the studied genes in the liver, spleen and lungs appeared only at the dose of 200 mg/kg bodyweight [140]. The most significant aberration in gene expression was observed in tissue with a high proliferation frequency, which complies with the elevated incidence of malignant neoplasms observed by Sofritti et al. According to these researchers, the consumption of aspartame may have carcinogenic effects [141].

Further evidence supporting the carcinogenic effects of aspartame was acquired in studies in rats from prenatal age to death: contact with aspartame at such an early stage of life appears to increase its carcinogenic effect [142]. In addition, semiquantitative PCR indicated an increase in P27 and H-ras gene expression in the liver tissue of rats fed with aspartame; a dose-dependent, significant increase in h-Ras gene expression was found for each treatment group compared to the control group. Moreover, the expression of P27 mRNA decreased in a manner dose-dependent manner compared to controls [143].

Pancreatic cancer belongs to the rarer neoplasms and since its late detection it usually has a high mortality rate [144]. However, aspartame was not found to influence the occurrence of pancreatic cancer in rodents. In fact, it is possible that using aspartame as a substitute for glucose may lower the risk of pancreatic cancers [145]. In addition, consumption of saccharine and other artificial or low caloric sweeteners (mainly aspartame) does not appear to influence the risk of pancreatic, gastric or endometrial cancers [146]. Furthermore, there is a relationship between consumption of sugars, which is indirectly related with consumption of sweeteners, and the risk of stomach and pancreatic cancer, but there is no relationship between consumption of sugars and risk of endometrial cancer [147,148,149].

In addition, in men, higher ADH activity, involved in the metabolism of aspartame, increased the risk of NHL and multiple myeloma. These findings support the opinion that aspartame is connected with elevated risk of NHL and multiple myeloma in men and with the increased risk of leukemia for males and females [138]. In contrast, McCullough et al. did not observe any associations between aspartame consumption and risk of all NHL, and only a few cases of rare NHL subtypes were seldom especially gender-specific types. Therefore determination of association of these types and aspartame is beyond belief [144]. Although studies performed on rodents in vivo and in vitro and research on the expression of proto-oncogenes and tumor suppressor genes suggest that aspartame may have carcinogenic properties [139,140,143], it is not possible to conclusively determine that aspartame is carcinogenic for humans. Indeed, most research fails to identify any such association [73,144].

## 14. Conclusions

The discovery of aspartame made it possible to replace sugar with a less caloric product. Safety studies have found the metabolic products of aspartame (aspartic acid, phenylalanine, and methanol) to be more harmful to the body than the original substance itself. Nevertheless, it is unclear whether aspartame is the direct cause of disease. There may be some connection between the consumption of aspartame and the development of DM and T2D. Some studies suggest that aspartame consumption has influence on obesity levels, glucose and insulin intolerance, and changes in the microbiota of the offspring of rats. In humans, there have been reports of premature birth as well as allergic reactions and weight gain in the newborns. Studies involving girls aged 9–10 have shown that aspartame increases the risk of an early first menstruation (<11 years).

Aspartame consumption can cause mood disorders, mental stress, and depression. Maternal absorption of aspartame during pregnancy correlates with autism in children. Long-term aspartame use influences the cerebral and cerebellar cortex: it can cause neurodegeneration, modify the functions of neuronal cells, interrupt homeostasis, learning and memory (Table 4).

Aspartame has been shown not to cause hypersensitivity even in a group of people with documented sensitivity. A few single cases have also been reported in which the withdrawal of aspartame-containing agents resulted in the disappearance of skin inflammation. In specific doses, aspartame appears to have analgesic and anti-inflammatory properties and it can contribute to the inhibition of ear inflammation caused by atopic dermatitis.

As an ingredient in many food products, aspartame, the metabolite of which is phenylalanine, is particularly harmful to people with phenylketonuria. During digestion, aspartame releases 50% of its mass in the form of phenylalanine, which leads to an increase in its level in the blood.

Although its genotoxicity is unknown, aspartame has elevated proliferation and slow apoptosis in test cells and could have carcinogenic properties. An increase in the markers Ki 67, PCNA and bcl-2 was noted. Studies on rats have found 200 mg/kg bodyweight to cause a significant increase in the markers c-myc, Ha-ras and the p53 suppressor gene. Exposure to aspartame from prenatal age, increases the incidence of lymphomas/leukemias in females. Studies have also confirmed an increase in P27 and H-ras expression. The relationship between aspartame and possible pancreatic, gastric, and endometrial cancer has not been proven.

Aspartame is a widely used sweetener both in the food and pharmaceutical industries. Knowledge about advantages and disadvantages of aspartame is crucial to assess the risk of its harmful impact on health. According to current knowledge benefits of aspartame use outweighs the possible side effects, hence this artificial sweetener remains basic excipient in products. Taking to account that aspartame is a widely used artificial sweetener, it seems appropriate to continue research on safety.

## 15. Methods

Due to widespread use of aspartame, articles used in this narrative review were searched regarding side effect of aspartame use and research in this field. The aim of this paper was to determine negative impact of aspartame on human body. Literature in this field was search using data bases provided by The Medical University of Lodz e.g., Science Direct, Scopus, Web of Science and looking for articles by entering specific phrases such as: aspartame, side effects of aspartame, aspartame genotoxicity, allergies and skin problems after aspartame administration, aspartame influence on obesity, aspartame impact on diabetes mellitus, aspartame cancer properties, aspartame metabolism. The most reliable publications and the most common side effects were selected. The most significant side effects were selected from found possible side effects caused by administration of aspartame.

## Figures and Tables

**Table 1:** Products of aspartame metabolism.

Aspartic Acid	Phenylalanine	Methanol (Metabolized into Formate and Formic Acid)
40%	50%	10%

**Table 2:** Influence on endocrine system.

Hormone	Result
cortysol	↑
corticosterone	↑
adrenocorticotropic hormone	↑

↑ increased level of hormone caused by use of of aspartame.

**Table 3:** Influence on nervous system.

Reaction after Aspartame Intake	Result
Increased cortisol level	Sympathetic nervous system is stimulated
Mental stress	Elevation of corticosterone and adrenocorticotropic hormone levels in plasma
Increased plasma Phy level	Decreased dopamine and serotonin levels in the brain (e.g., depression can occur)
Aspartame competition with glutamate to bind NMDA receptor	Neuronal cell function can be altered, memory loss
Calcium channels activation	Cell death
Amyloidogenic properties of aspartame	formation of Aβ-amyloid fibrils, which are associated with Alzheimer’s disease

**Table 4:** Effects of aspartame in various diseases.

Type of Disease	Influence of Aspartame
Obesity	It is unclear if obesity is associated with the consumption of products containing aspartame.
Diabetes mellitus	The connection between aspartame and TD2 risk is unclear.
Impact on children and fetus	Aspartame may have influence on children and fetuses.
Genotoxicity	Aspartame may have genotoxic properties.
Behavioral disorders	Aspartame can cause long-term changes in behavior.
Autism	Aspartame itself does not trigger autism.
Neurodegeneration	Aspartame cause mental stress, affects learning skills and memory. Aspartame is also amyloidogenic.
Neurotransmission	Aspartame reduces catecholamine levels.
Hormones	Aspartame elevates plasma corticosterone level and plasma adrenocorticotropic level.
Allergies and skin problems	Aspartame can induce systemic contact dermatitis (in huge daily doses which leads to formaldehyde accumulation). 0.5 μg kg^−1^ and 0.5 mg kg^−1^ doses of aspartame reduces some atopic dermatitis symptoms.
Phenylketonuria	Aspartame intake rises plasma Phe level. People suffering from phenylketonuria should avoid products containing aspartame.
Cancer	Aspartame may have carcinogenic properties but further studies are needed.

## Data Availability

Not applicable.
